# Ascorbic Acid Biosynthesis and Brackish Water Acclimation in the Euryhaline Freshwater White-Rimmed Stingray, *Himantura signifer*

**DOI:** 10.1371/journal.pone.0066691

**Published:** 2013-06-18

**Authors:** Samuel Z. H. Wong, Biyun Ching, You R. Chng, Wai P. Wong, Shit F. Chew, Yuen K. Ip

**Affiliations:** 1 Department of Biological Sciences, National University of Singapore, Kent Ridge, Singapore, Republic of Singapore; 2 Natural Sciences and Science Education, National Institute of Education, Nanyang Technological University, Singapore, Republic of Singapore; Universidade de Brasília, Brazil

## Abstract

L-gulono-γ-lactone oxidase (Gulo) catalyzes the last step of ascorbic acid biosynthesis, which occurs in the kidney of elasmobranchs. This study aimed to clone and sequence *gulonolactone oxidase* (*gulo*) from the kidney of the euryhaline freshwater stingray, *Himantura signifer*, and to determine the effects of acclimation from freshwater to brackish water (salinity 20) on its renal *gulo* mRNA expression and Gulo activity. We also examined the effects of brackish water acclimation on concentrations of ascorbate, dehydroascorbate and ascorbate + dehydroascorbate in the kidney, brain and gill. The complete cDNA coding sequence of *gulo* from the kidney of *H. signifer* contained 1323 bp coding for 440 amino acids. The expression of *gulo* was kidney-specific, and renal *gulo* expression decreased significantly by 67% and 50% in fish acclimated to brackish water for 1 day and 6 days, respectively. There was also a significant decrease in renal Gulo activity after 6 days of acclimation to brackish water. Hence, brackish water acclimation led to a decrease in the ascorbic acid synthetic capacity in the kidney of *H. signifer*. However, there were significant increases in concentrations of ascorbate and ascorbate + dehydroascorbate in the gills (after 1 or 6 days), and a significant increase in the concentration of ascorbate and a significant decrease in the concentration of dehydroascorbate in the brain (after 1 day) of fish acclimated to brackish water. Taken together, our results indicate that *H. signifer* might experience greater salinity-induced oxidative stress in freshwater than in brackish water, possibly related to its short history of freshwater invasion. These results also suggest for the first time a possible relationship between the successful invasion of the freshwater environment by some euryhaline marine elasmobranchs and the ability of these elasmobranchs to increase the capacity of ascorbic acid synthesis in response to hyposalinity stress.

## Introduction

Ascorbic acid is water-soluble and has a molecular mass of 176.1. At physiological pH, ascorbic acid is present mainly as the ascorbate anion [1]. Ascorbate functions mainly as a water-soluble antioxidant and a cofactor of enzymes involved in biosynthetic reactions [2]. Physiologically, ascorbate acts as a component of the intracellular antioxidant system which includes low molecular mass substances such as tocopherol and glutathione, and antioxidant enzymes such as superoxide dismutase and glutathione peroxidase [3]. Ascorbate is oxidized (one-electron transfer) to the ascorbyl free radical which can be further oxidized with the loss of a second electron to dehydroascorbate [4]. As an antioxidant, ascorbate protects tissues from oxidative damages by reacting with and inactivating radicals and oxidants such as superoxide anion, peroxyl- and hydroxyl-radicals, singlet oxygen, and hypochlorous acid [5], [6], [7], [8]. Moreover, ascorbate can regenerate α-tocopherol from α-tocopheryl radical in membranes [9].

Biosynthesis of ascorbic acid from glucose occurs in kidney of the majority of cold-blooded vertebrates and some birds, liver of some birds and the majority of mammals, and both kidney and liver of some birds and some marsupials. L-gulono-γ-lactone oxidase (GULO/Gulo) is the enzyme required for the last step of ascorbic acid biosynthesis, and it is expressed in kidney of elasmobranchs, some non-teleost fishes, amphibians, reptiles, non-passerine birds and monotremes, kidney and liver of some passerine birds and marsupials, and liver of some birds, some marsupials and most placental mammals [10]. Fishes like elasmobranchs, lampreys, sturgeons and lungfishes express Gulo and synthesize ascorbic acid in the kidney [10]. In general, Gulo/GULO is a microsomal enzyme that catalyzes the aerobic conversion of gulonolactone to ascorbate, with the production of hydrogen peroxide [11], [12]. Invertebrates, teleost fishes, some passerine birds, guinea pigs, bats and anthropoid primates, including humans, lack functional Gulo/GULO [13], [14], and therefore need a dietary intake of ascorbic acid as vitamin C. Although the evolution of ascorbic acid biosynthesis and the related kidney/liver transition have attracted great attention in the past, there is a paucity of molecular information on *gulo* from elasmobranchs, stingrays in particular [15]. Furthermore, it is unclear why elasmobranchs synthesize ascorbic acid, and there is a dearth of knowledge on effects of changes in environmental conditions (e.g. salinity changes) on *gulo* expression in elasmobranchs.

One of the main physiological functions of ascorbate is to maintain cellular homeostasis during stress [2]. Any environmental disturbance can be considered a potential source of stress, because the animal has to deal with the physiological changes triggered by changes in the environment. As has been demonstrated in turtle [16], [17], the greater the stress an animal undergoes, the more the ascorbic acid it has to produce or obtain from the diet. Salinity changes can cause stress; therefore, aquatic organisms have to deal with salinity stress, and ascorbate can be an important anti-stress agent. The branchial ascorbate concentration of the euryhaline mud crab, *Scylla serrata*, decreases with an increase in salinity indicating a possible role of ascorbate in combating stress in the gills [18]. Similarly, for the freshwater Philippine catfish, *Clarias batrachus*, which lacks the ability to synthesize ascorbic acid, ascorbate concentrations in the liver, kidney, brain and muscle decrease with increasing salinity [19]. More importantly, due to an increase in energy demand for osmoregulation, acclimation to different salinities can result in increased metabolic rate, which can in turn lead to increased production of reactive oxygen species. Indeed, it has been established that salinity changes result in oxidative stress in sea bass [20] and sturgeon [21]. However, at present, there is a dearth of knowledge on the effects of salinity changes on the concentrations of ascorbate, and its oxidative product, dehydroascorbate, in various organs/tissues of elasmobranchs which can synthesize ascorbic acid in kidney.

The majority of elasmobranchs are marine, and there are few stenohaline freshwater elasmobranchs. For a predominantly marine group with rare lineages that have successfully invaded and fully adapted to freshwater, euryhalinity is a relatively uncommon feature of elasmobranchs (as compared with teleosts), and there are only several notable examples such as *Carcharhinus leucas* and some populations of *Dasyatis sabina* [22]. The freshwater Asian white-rimmed stingray, *Himantura signifer* (Compagno and Roberts 1982), belongs to Family Dasyatidae, and can be found in the Batang Hari Basin in Jambi of Sumatra in Indonesia. Although *H. signifer* can be found in freshwater, it may re-enter brackish/estuarine and marine environments for reproduction [23]. Unlike the non-ureogenic stenohaline freshwater Amazonian stingray, *Potamotrygon motoro* [24], *H. signifer* is ureogenic and undergoes ureosmotic osmoregulation in brackish water [24]. Thus, *H. signifer* has to suppress urea synthesis and retention in freshwater, but it cannot stop urea synthesis completely. As a result, the plasma and tissues of freshwater *H. signifer* contain higher concentrations of urea than other freshwater fishes [24]. Because of this, *H. signifer* could be exposed to greater salinity stress in freshwater than in brackish water. Since salinity stress is known to induce oxidative stress in fish [18], [19], *H. signifer* could be exposed to greater salinity-induced oxidative stress in freshwater than in brackish water. Furthermore, since *H. signifer* is nitrogen-limited, and conserves dietary nitrogen as urea for osmoregulatory purposes [25], it is possible that it would be confronted with postprandial osmotic stress resulting from increased urea synthesis upon feeding in freshwater.

Therefore, this study was undertaken to obtain the full cDNA sequence of *gulo* from the kidney of *H. signifer*, and to determine its mRNA expression in the kidney of fish kept in freshwater (control) or exposed to brackish water (salinity 20) for 1 or 6 days using quantitative RT-PCR. Since *H. signifer* could be exposed to decreased salinity stress in brackish water, we aimed to test the hypothesis that exposure to brackish water would result in a down-regulation of its renal *gulo* expression. In order to confirm that salinity changes indeed affected ascorbic acid synthesis in *H. signifer*, efforts were made to determine the renal Gulo activity and the concentrations of ascorbate and dehydroascorbate in the kidney, brain and gills of *H. signifer* exposed to freshwater or brackish water. The hypothesis tested was that, despite a decrease in renal Gulo activity, acclimation from freshwater to brackish water would lead to increases and decreases in the concentrations of ascorbate and dehydroascorbate, respectively, in some organs of *H. signifer*, reflecting the redox status due to a decrease in salinity-induced oxidative stress in brackish water.

## Materials and Methods

### Ethics statement

This study was approved by the Institutional Animal Care and Use Committee of the National University of Singapore (IACUC 021/10). In Singapore, no specific permission from an authority is required for collection of seawater. The current study is not an ecological/field study, and no animals were collected from the field.

### Animals

Specimens of *H. signifer* were purchased from a local fish farm. Fish were kept in dechlorinated tap water (freshwater; pH 6.8–7.0) at 25°C in plastic tanks of appropriate sizes with aeration under a 12 h light∶12 h dark regime for at least 1 week before experiments. They were fed live shrimps daily.

### Experimental conditions and collection of samples

Control fish (N = 5) were immersed in 25 volumes (v/w) of freshwater in plastic tanks, and they served as controls for the two experimental conditions: exposure to brackish water (salinity 20; pH 7.8) for 1 or 6 days. For exposure to salinity changes, fish (N = 5) were transferred from freshwater (day 0) to waters of salinity 5 on day 1, salinity 10 on day 2, salinity 15 on day 3, salinity 20 on day 4 and kept in salinity 20 for 1 or 6 days. Water was changed daily. Natural seawater was collected from the sea at least 1 km away from the coast of the Singapore main island. Waters of different salinities were prepared by mixing seawater with an appropriate quantity of freshwater. Salinity was monitored using a YSI Model 30/10 FT salinometer (Yellow Springs Instrument Co. Inc, Ohio, USA). During salinity acclimation, stingrays were fed live shrimps on alternate days. Both control and experimental fish were killed by an overdose of neutralized 0.05% MS222, and their tissues quickly excised, frozen in liquid nitrogen and stored at −80°C until analysis.

### Total RNA extraction and cDNA synthesis

Total RNA was isolated from kidney samples of *H. signifer* using TRI REAGENT™ protocol and purified using the Qiagen RNeasy Mini Kit (Qiagen GmbH, Hilden, Germany). The RNA was quantified spectrophotometrically using Hellma TrayCell (Hellma GmbH & Co. KG, Müllheim, Germany) and its integrity checked electrophoretically to verify RNA by comparing the 18S and the 28S bands, which were visualized by a G:Box gel documentation system (Syngene, Cambridge, UK). Total RNA (1 µg) isolated was reverse transcribed into cDNA using RevertAid™ First Strand cDNA synthesis kit (Fermentas International, Inc, Burlington, ON, Canada).

### Polymerase Chain Reaction (PCR)

The complete *gulo* sequence was obtained using primers (Forward: 5′- TYC TCM AGG TGG AYM WGG AGA -3′; Reverse: 5′- TCR STG TGW GGR AAC CAG A -3′) designed from the conserved regions of *Triakis scyllium* (ABO15547.1), *Mustelus manazo* (ABO15548.1), *Scyliorhinus torazame* (Q90YK3.1), *Acipenser transmontanus* (ABO15549.1), *Rattus norvegicus* (NM_022220), *Mus musculus* (AY453064) and *Sus scrofa* (AF440259), as reported in Cho et al. [15]. PCR was carried out in Biorad Peltier thermal cycler (Biorad, Hercules, CA, USA) using DreamTaq™ DNA polymerase (Fermentas International Inc.). The cycling conditions were 94°C (3 min), followed by 35 cycles of 94°C (30 s), 55°C (30 s), 72°C (2 min) and 1 cycle of final extension at 72°C (10 min). PCR products were electrophoresed in 1% agarose gel. Bands of the estimated size were extracted from the gels using QIAquick® Gel Extraction Kit (Qiagen GmbH). Purified PCR products were subjected to cycle sequencing using BigDye® Terminator v3.1 Cycle Sequencing Kit (Applied Biosystems, Foster City, CA, USA) and purified by ethanol/sodium acetate precipitation. Purified products were automatically sequenced using the 3130XL Genetic Analyzer (Applied Biosystems). The fragments were verified to be *gulo* from Genbank database.

### Rapid amplification of cDNA ends (RACE)-PCR

Total RNA (1 µg) was reverse transcribed into 5′-RACE-Ready cDNA and 3′RACE-Ready cDNA using SMARTer™ RACE cDNA Amplification kit (Clontech Laboratories, Mountain View, CA, USA). RACE-PCR was performed using the Advantage® 2 PCR kit (Clontech Laboratories) to generate the 5′ and 3′ cDNA fragments, with 5′- GAA TGC TCG GAG TCA GTA AAC CGG G -3′ and 5′- GGT TCT AAC CGT CAC CAT CCA GT -3′, respectively. RACE-PCR cycling conditions were 25 cycles of 94°C for 30 s, 65°C for 30 s and 72°C for 4 min. RACE-PCR products were separated using gel electrophoresis, purified and sequenced. Multiple sequencing was performed in both directions to obtain the full-length cDNA. Sequence assembly and analysis were performed using BioEdit [26]. The cDNA sequence of *gulo* from *H. signifer* has been deposited to the Genbank with accession number (KC465465).

The Gulo amino acid sequence was translated from the nucleotide sequence of *gulo* using ExPASy Proteomic server [27]. The potential phosphorylation, O-GlcNAcylation and N-GlcNAcylation sites were predicted using NetPhos 2.0 [28], YinOYang 1.2 [29], [30] and NetNglyc 1.0 [31], respectively. The transmembrane regions (TMs) were predicted using MEMSATS & MEMSAT-SVA provided by PSIPRED protein structure prediction server (http://bioinf.cs.ucl.ac.uk/psipred/) [32].

### Phylogenetic analysis

Amino acid sequences of Gulo/GULO from other animals were obtained from Genbank with the following accession numbers: *Triakis scyllium* (ABO15547.1), *Mustelus manazo* (ABO15548.1), *Scyliorhinus torazame* (Q90YK3.1), *Acipenser transmontanus* (ABO15549.1), *Raja kenojei* (EF397523.1), *Dasyatis akajei* (EF397524.1), *Xenopus laevis* (NM_001095065), *Gallus gallus* (XM_001234313), *Meleagris gallopavo* (XM_003204567.1), *Pelodiscus sinensis* (AET14634.1), *Bos taurus* (Q3ZC33.3), *Sus scrofa* (NP_001123420.1), *Hipposideros armiger* (ADP88814.1), *Rousettus leschenaultii* (ADP88813.1), *Rattus norvegicus* (P10867.3), *Mus musculus* (P58710.3), *Ornithorhynchus anatinus* (XM_001521551), and *Lysinibacillus sphaericus* (YP_001699013.1). These sequences were aligned using ClustalX2 and phylogenetic analysis was performed using neighbor-joining method and 100 bootstrap replicates with Phylip [33].

### Tissue expression

The mRNA expression of *gulo* was performed on twelve different organs/tissues, including brain, heart, eye, spleen, stomach, intestine, kidney, gills, skin, liver and muscle. PCR was carried out in Biorad Peltier thermal cycler (Biorad, Hercules) using DreamTaq™ DNA polymerase (Fermentas International Inc.) and using forward primer 5′- TYC TCM AGG TGG AYM WGG AGA -3′ and reverse primer 5′- TCR STG TGW GGR AAC CAG A -3′. The cycling conditions consist of 95°C (3 min), followed by 30 cycles of 94°C (30 s), 60°C (30 s), 72°C (30 s) and 1 cycle of final extension at 72°C (10 min). PCR products were then electrophoresed in 2% agarose gel.

### qPCR

RNA from kidney samples were treated with Deoxyribonuclease I, (Sigma–Aldrich Co., St Louis, MO, USA) to remove any contaminating genomic DNA. First strand cDNA was then synthesized from 1 µg of total RNA using random hexamer primer and the RevertAid™ first stand cDNA synthesis kit (Fermentas International Inc.). qPCR was performed in triplicates using a StepOnePlus™ Real-Time PCR System (Applied Biosystems). The standard cDNA (template) was serially diluted in sterile water (from 10^7^ to 10^2^ specific copies/2 µl). The PCR reactions contained 5 µl of 2× Fast SYBR® Green Master Mix (Applied Biosystems), 0.1 µM of forward (5′- CCT CAC ACG GAC AAG ACA G -3′) or reverse (5′- CTG GAA CTG CTG TTG TGG A -3′) qPCR primers, and cDNA (equivalent to 1 ng of RNA) or standard (2 µl) in a total volume of 10 µl. Cycling conditions were 95°C for 20 s (1 cycle), followed by 45 cycles of 95°C for 3 s and 60°C of 30 s. Data (C_T_ values) were collected at each elongation step. Runs were followed by melt curve analysis by increasing from 60°C to 95°C in 0.3°C increments to confirm the presence of only a single product. The PCR products were separated in a 2% agarose gel to verify the presence of a single band.

In order to determine the absolute quantity of *gulo* transcripts in a qPCR reaction, efforts were made to produce a pure amplicon of a defined region of *gulo* cDNA from the kidney of *H. signifer* following the methods of Gerwick et al. [34]. PCR was performed with qPCR primers and cDNA as a template in a final volume of 25 µl with the following cycling conditions: initial denaturation 95°C for 3 min, followed by 35 cycles of 95°C for 30 s, 60°C for 30 s and 72°C for 30 s and 1 cycle of final extension of 72°C for 10 min. PCR product was separated in a 2% agarose gel. The product was excised and purified using QIAquick gel extraction kit (Qiagen GmbH). The *gulo* nucleotide fragment in the purified product was cloned using pGEM®-T Easy vector (Promega Corporation, Madison, WI, USA). The presence of the insert in the recombinant clones was confirmed by sequencing. The cloned circular plasmid was quantified using a spectrophotometer. Copy numbers were calculated from the C_T_ values of the standards [33]. A standard curve was obtained from plotting threshold cycle (C_T_) on the Y-axis and the natural log of concentration (copies µl^−1^) on the X-axis. The C_T_, slope, PCR efficiency, Y-intercept and correlation coefficient (R^2^) were calculated using the default setting of StepOne™ Software v2.1 (Applied Biosystems). Diluted standards were stored at −20°C. The PCR efficiency for *gulo* was 91.6%. The quantity of transcript in an unknown sample was determined from the linear regression line derived from the standard curve and the number of copies per ng cDNA.

### Determination of specific activity of Gulo

Specific activity of Gulo from the kidney of *H. signifer* was measured using a modification of the direct spectrophotometric assay described by Dabrowski [35]. Kidney samples from *H. signifer* were homogenized in 5 volumes (w/v) of homogenization buffer containing 50 mM NaHPO_4_ buffer (pH 7.4), 0.2% Na-deoxycholate and 1 mM EDTA three times using an Ultra-Turrax T25 homogenizer (Ika®-Labortechnik, Staufen, Germany) at 24,000 r.p.m. for 20 s each with 10 s intervals. Homogenates were centrifuged at 10,000×*g* for 30 min at 4°C to obtain the supernatants, which were used for determination of specific activity of Gulo at 25°C. The assay medium contained 100 mM L-gulonolactone, 50 mM reduced glutathione in 50 mM NaHPO_4_ buffer (pH 7.4). The reactions were stopped after 0 or 2 h by addition of ice-cold 10% metaphosphoric acid to the reaction mixtures. The reaction mixtures were then centrifuged at 10,000×*g* for 15 min at 4°C to obtain the supernatants, which were used directly for determination of concentrations of (ascorbate + dehydroascorbate) using a commercial Vitamin C assay kit (Cosmo Bio Co., Ltd., Tokyo, Japan). Specific activities of Gulo were expressed as µg ascorbic acid formed h^−1^ g^−1^ wet mass or µg ascorbic acid formed h^−1^ mg^−1^ protein.

### Determination of concentrations of ascorbate and dehydroascorbate

Samples of the brain, kidney and gills of *H. signifer* were homogenized in 13 volumes (w/v) of ice-cold 5.4% metaphosphoric acid three times using an Ultra-Turrax T25 homogenizer (Ika®-Labortechnik) at 24,000 r.p.m. for 20 s each with 10 s intervals. The homogenates were centrifuged at 10,000×*g* for 30 min at 4°C to obtain the supernatants, which were used directly for the determination of concentrations of (ascorbate + dehydroascorbate), and dehydroascorbate alone, using a commercial Vitamin C Assay Kit (Cosmo Bio Co., Ltd., Tokyo, Japan). Total (ascorbate + dehydroascorbate) measurement involved the addition of an oxidizing reagent to the reaction, according to the manufacturer's protocol, while measurement of dehydroascorbate excludes this step. L-ascorbic acid solution was freshly prepared, using 5% metaphosphoric acid, as a standard for comparison. The standard curve was linear in the range of 0.5 to 4 µg of ascorbic acid. Ascorbate concentrations were calculated from the differences between concentrations of ascorbate + dehydroascorbate and dehydroascorbate. Concentrations were calculated and expressed in µg g^−1^ wet tissue mass. Preliminary results indicated that the muscle ascorbate + dehydroascorbate concentration was close to the detection limit of the assay method and therefore not investigated in this study.

### Determination of thiobarbituric acid reactive substances

Thiobarbituric acid reactive substances were quantified as an index of lipid peroxidation [36] to elucidate the effect of brackish water acclimation on the redox-status of the gills and liver of *H. signifer*. These two organs were chosen because gills are in direct contact with the external medium and affected instantly by salinity changes, and the liver is the site of increased urea synthesis through the ornithine-urea cycle in response to brackish water acclimation. The frozen sample was homogenized three times, in 20 volumes (w/v) of ice-cold 1.1% phosphoric acid, for 20 s each with 10 s intervals using an Ultra-Turrax T25 homogenizer (Ika®-Labortechnik) at 24,000 r.p.m. Then, 0.4 ml of the homogenate was added to an equal volume of a mixture of 1% thiobarbituric acid, 0.1 mmol l^−1^ butylated hydroxytoluene solution, 50 mmol l^−1^ sodium hydroxide, and 0.2 ml of 7% phosphoric acid. After heating for 15 min at 98°C, the sample was vigorously mixed with 1.5 ml of butanol, and centrifuged at 2000×*g* for 5 min using a Beckman J2-21/E centrifuge (Beckman Coulter Inc., Fullerton, CA, USA). The organic layer was transferred to glass cuvettes, and the optical densities at 532 nm and 600 nm were determined using a Shimadzu UV-160A spectrophotometer (Shimadzu, Kyoto, Japan). Blanks were prepared by replacing the thiobarbituric acid with 3 mmol l^−1^ hydrochloric acid. The results were calculated as Sample (A_532_–A_600_) – Blank (A_532_–A_600_) as recommended by Ramos and Hermes-Lima [37]. TBARS values were obtained by using the extinction coefficient of 156 l mmol^−1^ cm^−1^.

### Statistical analysis

Results are presented as means ± standard errors of the mean (S. E. M.). Differences between means are evaluated using one-way analysis of variance (ANOVA), followed by the Tukey post-hoc test for liver and kidney data, and the Dunnett T3 post-hoc test for gill. Differences were regarded as statistically significant at *P*<0.05.

## Results

### Nucleotide sequences, translated amino acid sequences and phylogenetic analysis

**Figure 1:**
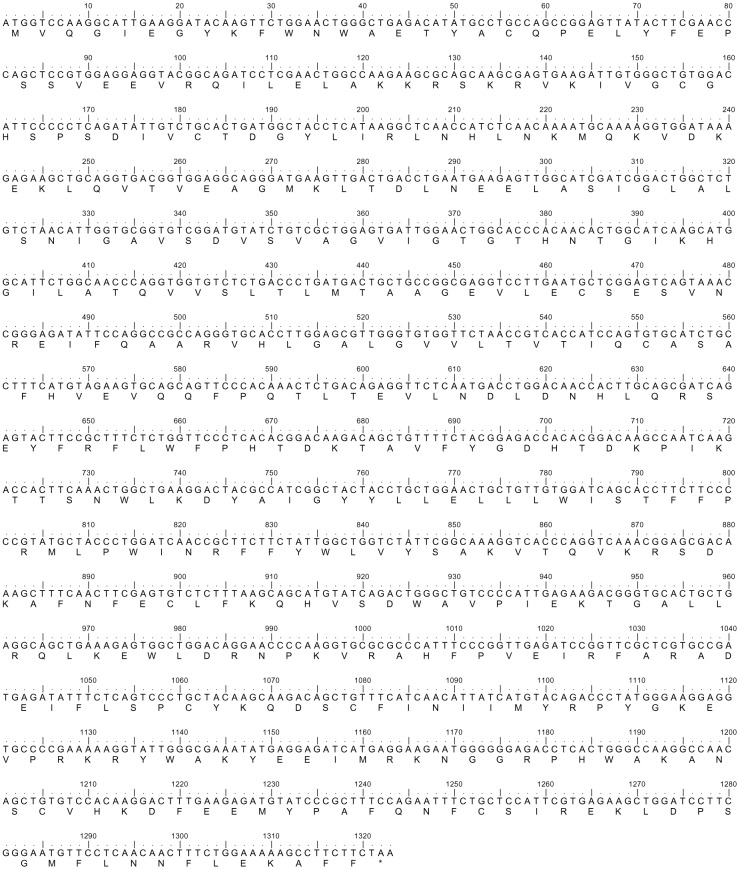
The cDNA sequence (GenBank accession number KC465465) of *gulonolactone oxidase* (*gulo*) and the deduced amino acid sequence of gulonolactone oxidase (Gulo) from the kidney of *Himantura signifer*.

**Figure 2:**
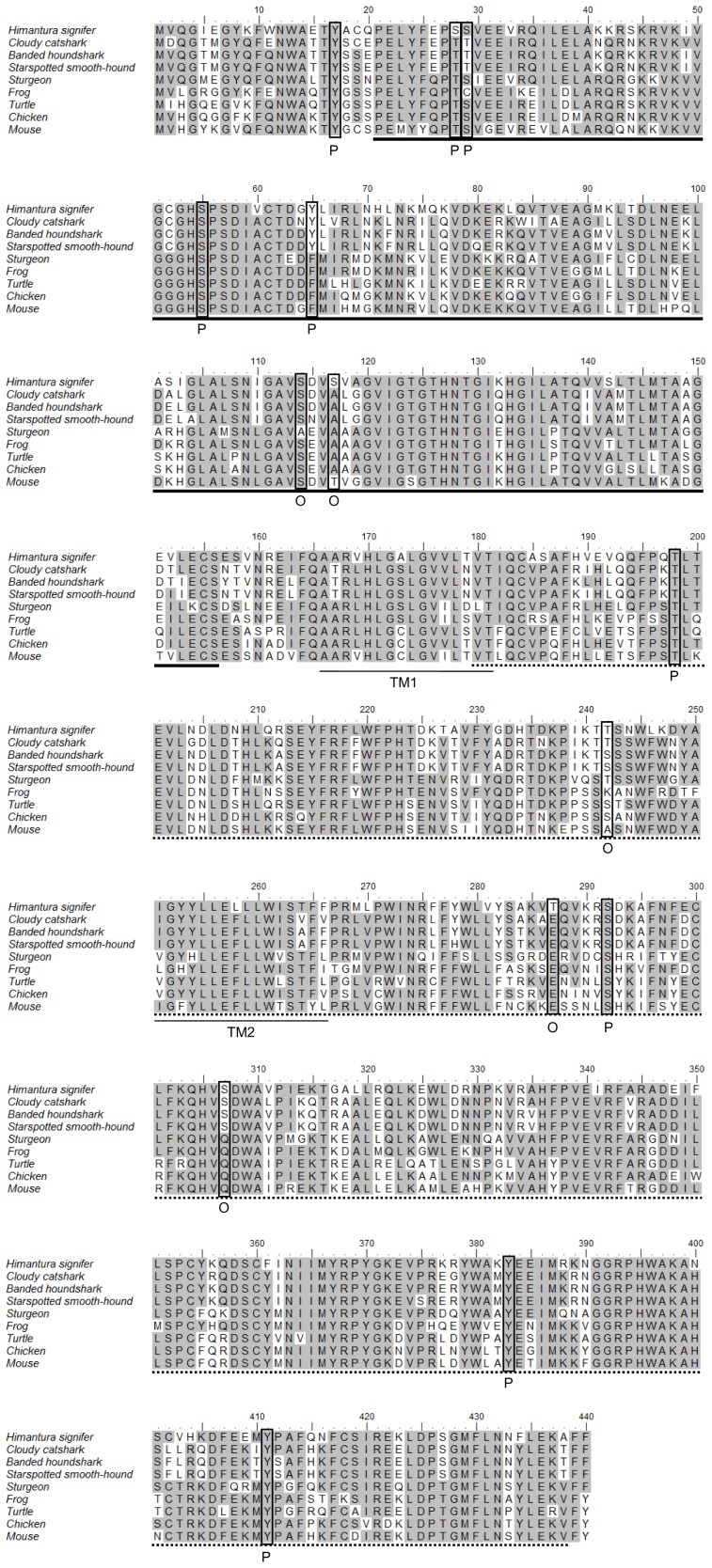
Multiple amino acid alignment of the deduced amino acid sequence of gulonolactone oxidase (Gulo) from the kidney of *Himantura signifer*, with eight other known sequences of Gulo from GenBank; *Scyliorhinus torazame* (cloudy catshark Gulo; Q90YK3.1), *Triakis scyllium* (banded houndshark Gulo; ABO15547.1), *Mustelus manazo* (starspotted smooth-hound Gulo; ABO15548.1), *Acipenser transmontanus* (sturgeon Gulo; ABO15549.1), *Xenopus laevis* (frog GULO; NM_001095065), *Pelodiscus sinensis* (turtle GULO; AET14634.1), *Gallus gallus* (chicken GULO; XM_001234313), and *Mus musculus* (mouse GULO; P58710.3). Identical amino acids are indicated by shaded residues. The FAD-binding and ALO domains are underlined in bold and dotted lines respectively. P denotes potential phosphorylation sites. O denotes potential O-GlcNAcylation sites. The predicted transmembrane domains are underlined. The transmembrane domains (TM) of Gulo of *H. signifer* were predicted using MEMSATS & MEMSAT-SVA provided by PSIPRED protein structure prediction server.

**Figure 3:**
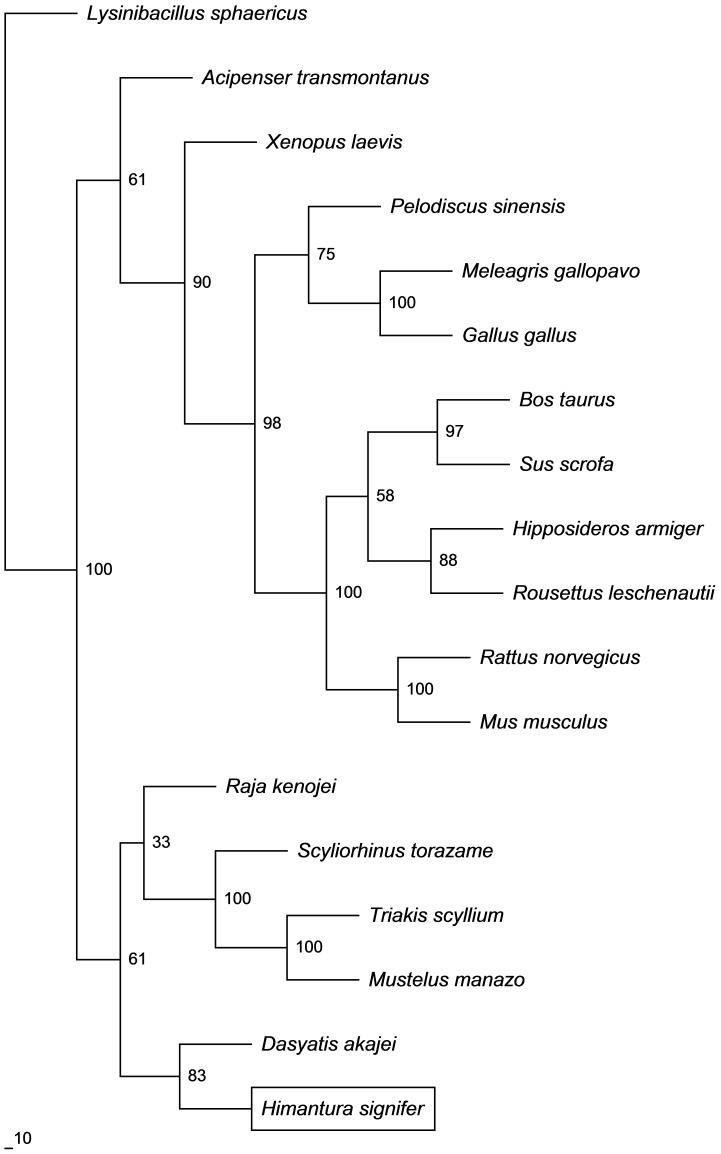
A phylogenetic tree that illustrates the relationship between the deduced amino acid sequence of gulonolactone oxidase (Gulo) of *Himantura signifer* and those of other animals with that of *Lysinibacillus sphaericus* as the outgroup.

The complete coding cDNA sequence of *gulo* from the kidney of *H. signifer* consisted of 1323 bp (Genbank accession number KC465465), which coded for 440 amino acids (Figure 1) with a calculated molecular mass of 50.8 kDa. The deduced amino acid sequence consisted of two conserved domains, the flavin adenine dinucleotide (FAD)-binding domain at positions 21–156 and the D-arabinono-1,4-lactone oxidase (ALO) domain at positions 180–438 (Figure 2). The percentage similarities between the amino acid sequence of Gulo from *H. signifer* and those from other animals ranged between 66.3 and 75.9 for fish species, and 46.2 and 67.5 for non-fish species (Table 1). An analysis of the number of phosphorylation and glycosylation sites of *H. signifer* compared with various animals revealed 9 phosphorylation and 5 O-GlcNAcylation sites (Table 2). In addition, a phylogenetic analysis confirmed that Gulo of *H. signifer* was closely related to those of other elasmobranchs (Figure 3).

**Table 1:** The percentage similarity between the deduced amino acid sequence of gulonolactone oxidase (Gulo) from the kidney of *Himantura signifer* and those of other animal species obtained from Genbank.

	Animal species	Similarity with Gulo from *H. signifer*
Fish	*Triakis scyllium*	75.9%
	*Mustelus manazo*	74.7%
	*Scyliorhinus torazame*	74.3%
	*Acipenser transmontanus*	66.3%
Amphibian	*Xenopus laevis*	67.5%
Bird	*Gallus gallus*	65.9%
	*Meleagris gallopavo*	65.9%
Reptile	*Pelodiscus sinensis*	63.1%
Mammal	*Bos taurus*	64.7%
	*Sus scrofa*	63.8%
	*Mus musculus*	63.4%
	*Rattus norvegicus*	63.4%
	*Rousettus leschenaultii*	62.2%
	*Hipposideros armiger*	61.8%
	*Ornithorhynchus anatinus*	46.2%

Sequences are arranged in descending order of similarities between groups and within the group of animals.

**Table 2:** Number of phosphorylation, O-GlcNAcylation and N-GlcNAcylation sites in the deduced amino acid sequence of gulonolactone oxidaseof various animals compared with *Himantura signifer*.

Species	Phosphorylation sites	O-GlcNAcylation sites	N-GlcNAcylation sites	Reference
*Himantura signifer*	9	5	0	This study
*Potamotrygon motoro*	9	3	0	Wong SZH, Ching B, Ip YK (unpublished results)
*Triakis scyllium*	20	5	1	[15]
*Mustelus manazo*	20	5	1	[15]
*Scyliorhinus torazame*	15	1	1	[38]
*Acipenser transmontanus*	20	3	0	[15]
*Xenopus laevis*	21	6	2	[68]
*Pelodiscus sinensis*	22	9	2	[69]
*Gallus gallus*	14	5	2	[70]
*Mus musculus*	18	7	2	[71]

A close examination of the alignment of Gulo from *H. signifer* with those from three species of shark, sturgeon, frog, turtle, chicken and mouse revealed that a considerable number of amino acid residues were unique to fish (Figure 2). For example, all the fish species including *H. signifer* have Gln3, Gln35, Glu38, Ala381 and Glu385, but all non-fish species have correspondingly Leu/His3, Glu35, Asp/Ala38, Val/Pro/Leu381 and Asn/Ser/Gly/Thr385 instead. Furthermore, all the fish Gulo sequences have Phe at C-terminus while non-fish sequences have Tyr. In addition, certain characters, which include Cys52, Tyr65, Asp225, Lys226, Thr241 and Ser307, were unique to all the four elasmobranch species compared to non-elasmobranchs (correspondingly Gly52, Phe65, Glu225, Asn226, Ser241 and Gln307). Apart from these shared features, *H. signifer* showed considerable unique amino acid residues in the conserved regions not found in the Gulo/GULO sequences of other vertebrates. Significant amino acid replacements might include Ser28, Lys42, Gln76, Ile103 in the FAD-binding domain and Ala185, Leu319, Glu325, Lys382 in the ALO domain.

### Tissue expression of *gulo*

**Figure 4:**
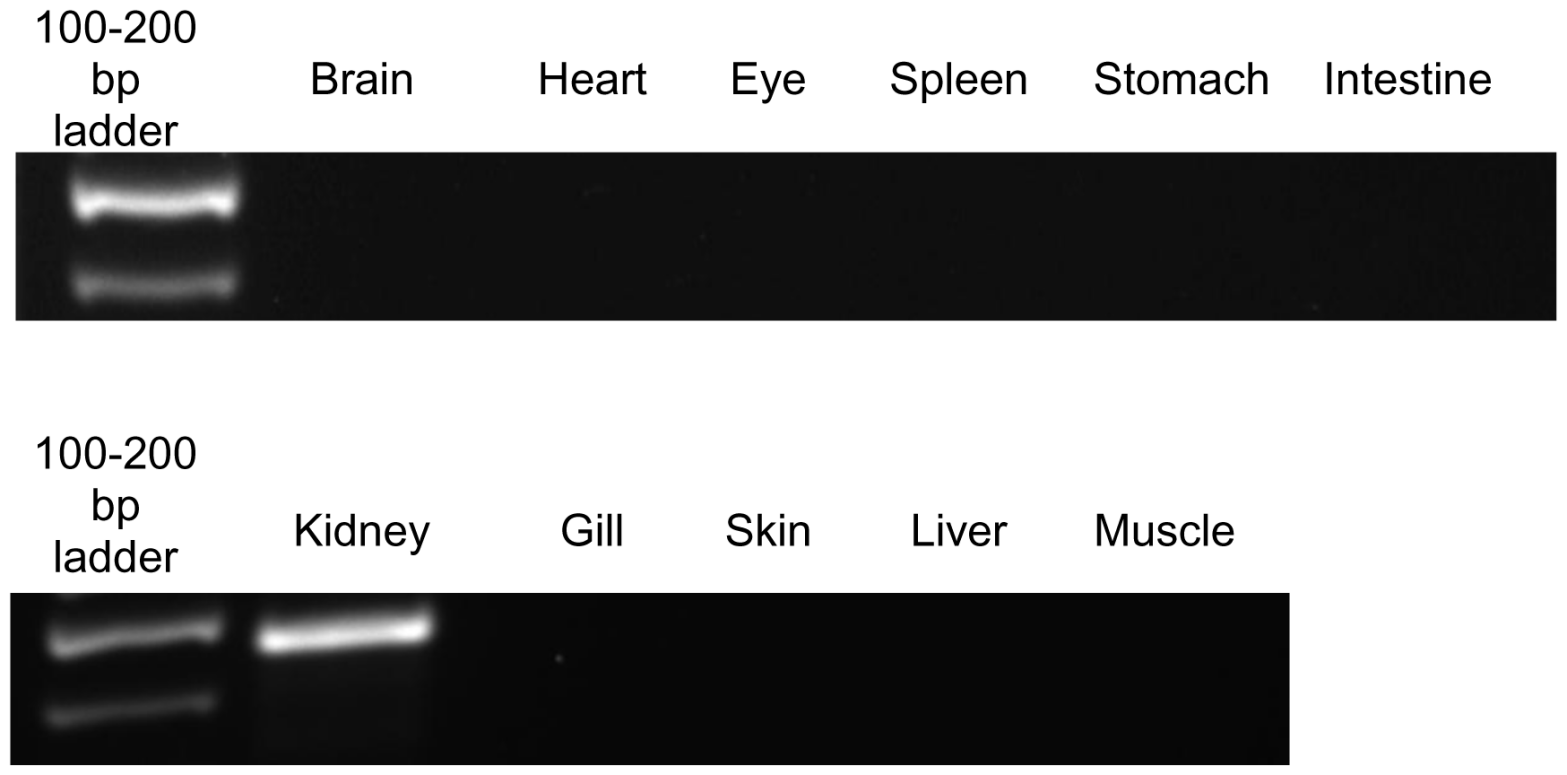
mRNA expression of *gulonolactone oxidase* (*gulo*) from the brain, heart, eye, spleen, stomach, intestine, kidney, gills, skin, liver and muscle of *Himantura signifer* kept in freshwater.

The mRNA expression of *gulo* in *H. signifer* was kidney-specific; it was not detectable in the brain, heart, eye, spleen, stomach, intestine, gills, skin, liver and muscle of fish kept in freshwater (Figure 4).

### mRNA expression of *gulo* from the kidney of fish exposed to brackish water

**Figure 5:**
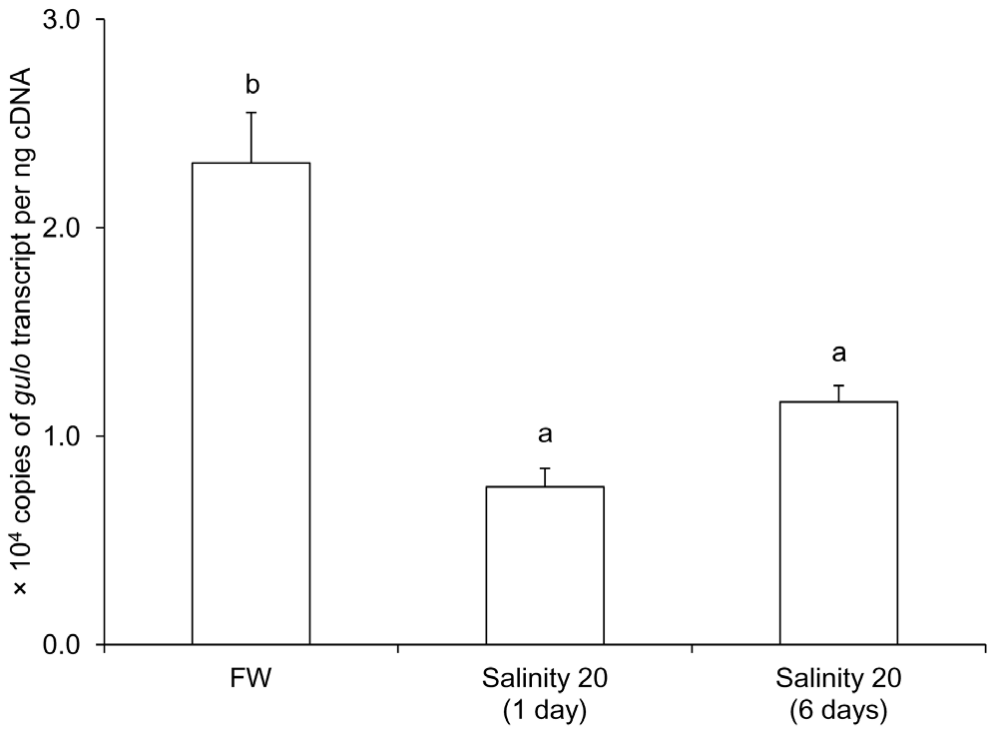
Absolute quantification (copies of transcript per ng cDNA) of mRNA expression of *gulonolactone oxidase* (*gulo*) from the kidney of *Himantura signifer* kept in freshwater (FW; control) or exposed to brackish water (salinity 20) for 1 or 6 days after a 4-day progressive increase in salinity. Results represent mean ± S. E. M. (N = 5). Means not sharing the same letter are significantly different (*P*<0.05).

There were significant decreases in the mRNA expression of *gulo* in the kidney of *H. signifer* after 1 day (by 67%) or 6 days (by 50%) of exposure to brackish water as compared to the freshwater control (Figure 5).

### Specific activity of Gulo from the kidney of fish exposed to brackish water

**Figure 6:**
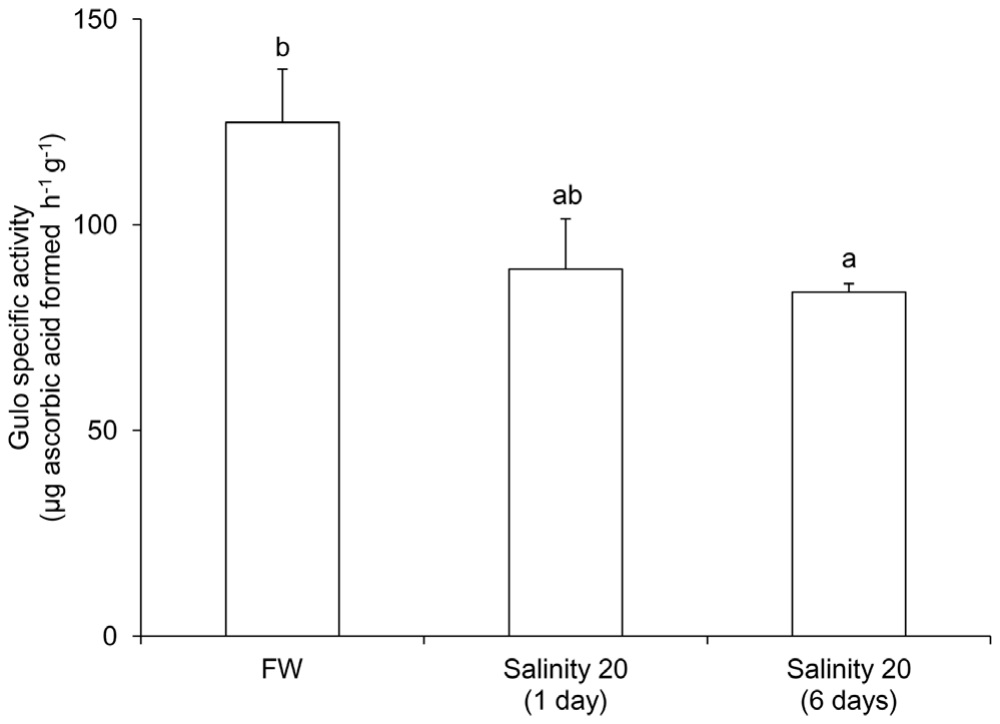
Specific activity of gulonolactone oxidase (Gulo; µg ascorbic acid formed h^−1^ g^−1^) from the kidney of *Himantura signifer* kept in freshwater (FW) on day 0 (control), or exposed to brackish water (salinity 20) for 1 or 6 days after a 4-day progressive increase in salinity. Results represent mean ± S. E. M. (N = 4). Means not sharing the same letter are significantly different (*P*<0.05).

The specific activity (µg ascorbic acid formed h^−1^ g^−1^) of Gulo from the kidney of *H. signifer* kept in freshwater was 125±12.9 (Figure 6). Although the renal Gulo activity was unaffected by 1 day of exposure to brackish water, it decreased significantly in fish exposed to brackish water for 6 days.

### Ascorbate and dehydroascorbate concentrations in the kidney, brain and gills of fish exposed to brackish water

**Figure 7:**
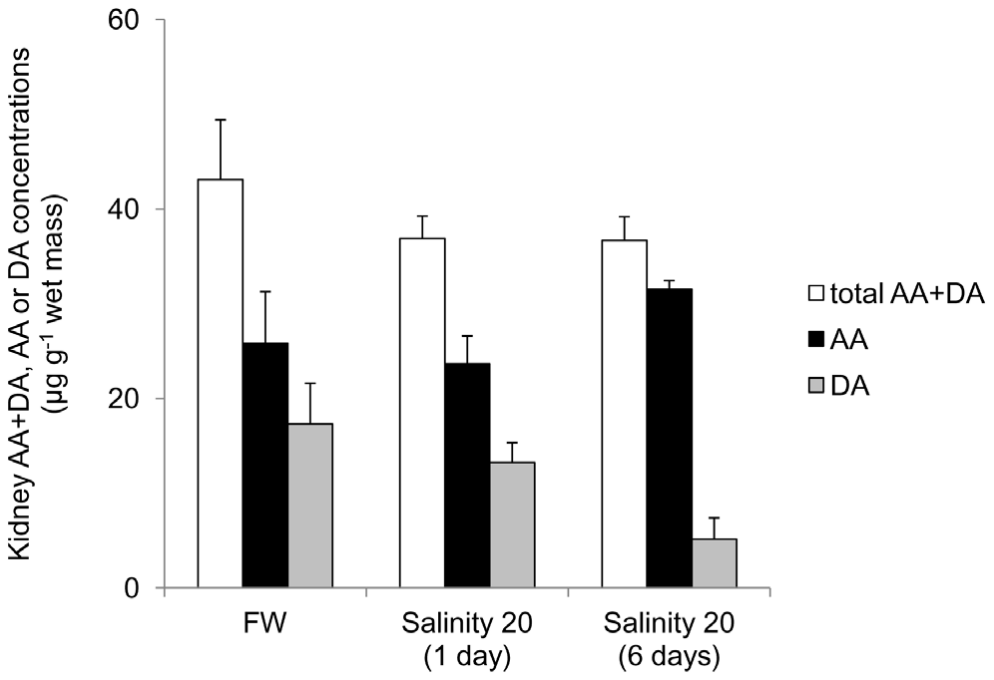
Concentrations (µg g^−1^ wet mass) of ascorbate (AA; ▪), dehydroascorbate (DA; ▪) or AA+DA (TAA; □) in the kidney of *Himantura signifier* kept in freshwater (FW) on day 0 (control), or exposed to brackish water (salinity 20) for 1 or 6 days after a 4-day progressive increase in salinity. There was no statistical difference between different groups. Results represent mean ± S. E. M. (N = 4).

The concentrations (µg g^−1^ wet mass) of ascorbate, dehydroascorbate and ascorbate + dehydroascorbate in the kidney of *H. signifer* kept in freshwater were 25.8±4.32, 17.3±5.45 and 43.1±6.31, respectively (Figure 7), and they remained unchanged in fish exposed to brackish water for 1 or 6 days.

**Figure 8:**
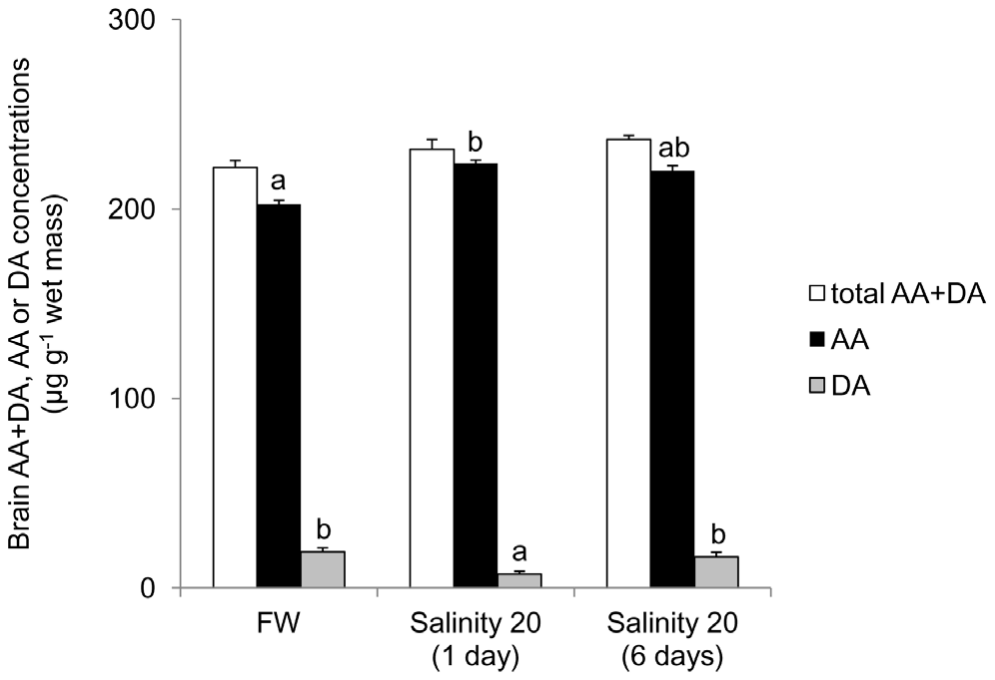
Concentrations (µg g^−1^ wet mass) of ascorbate (AA; ▪), dehydroascorbate (DA; ▪) or AA+DA (TAA; □) in the brain of *Himantura signifer* kept in freshwater (FW) on day 0 (control), or exposed to brackish water (salinity 20) for 1 or 6 days after a 4-day progressive increase in salinity. Results represent mean ± S. E. M. (N = 4). Means not sharing the same letter are significantly different (*P*<0.05).

The concentrations (µg g^−1^ wet mass) of ascorbate, dehydroascorbate and ascorbate + dehydroascorbate in the brain of *H. signifer* kept in freshwater were the highest among the three organs studied (203±4.07, 19.2±1.94 and 222±3.86, respectively; Figure 8). For fish exposed to brackish water for 1 day, there were a significant increase in the ascorbate concentration (by 1.1-fold) and a significant decrease in the dehydroascorbate concentration (by 62%) in the brain.

**Figure 9:**
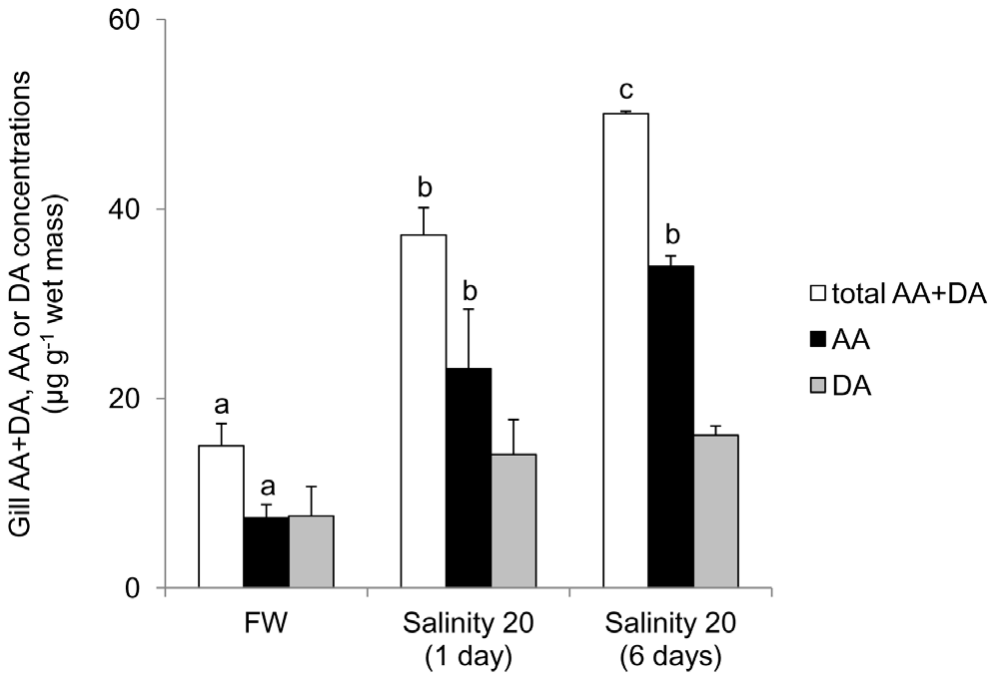
Concentrations (µg g^−1^ wet mass) of ascorbate (AA; ▪), dehydroascorbate (DA; ▪) or AA+DA (TAA; □) in the gills of *Himantura signifer* kept in freshwater (FW) on day 0 (control), or exposed to brackish water (salinity 20) for 1 or 6 days after a 4-day progressive increase in salinity. Results represent mean ± S. E. M. (N = 4). Means not sharing the same letter are significantly different (*P*<0.05).

The concentrations (µg g^−1^ wet mass) of ascorbate, dehydroascorbate and ascorbate + dehydroascorbate in the gills of *H. signifer* kept in freshwater were the lowest among the three organs studied (7.40±1.42, 7.61±3.10 and 15.0±2.36, respectively; Figure 9). After 1 day of exposure to brackish water, there was a significant increase in ascorbate concentration (by 3.1-fold), and in ascorbate + dehydroascorbate concentration (by 2.5-fold) in the gills. For fish exposed to brackish water for 6 days, concentrations of ascorbate and ascorbate + dehydroascorbate increased significantly by 4.6-fold and 3.3-fold, respectively.

### Products of lipid peroxidation in the gills and liver of fish exposed to brackish water

The concentrations (nmol g^−1^ wet mass ±S. E. M.; N = 4) of thiobarbituric acid reactive substances in the liver of *H. signifer* exposed to brackish water for 1 day (33.9±7.72) or 6 days (45.5±4.79) were not significantly different from that of the freshwater control (42.7±2.23). Similarly, the concentrations of thiobarbituric acid reactive substances in the gills of fish exposed to brackish water for 1 day (18.9±1.0) or 6 days (14.0±0.5) were not significantly different from that of the freshwater control (14.0±1.8).

## Discussion

### Molecular characterization of *gulo*/Gulo from the kidney of *H. signifer*

Similar to other elasmobranchs, e.g. *Raja kenojei* and *Dasyatis akajei* [15], *gulo* was expressed only in the kidney of *H. signifer*. The cDNA sequence of *gulo* from the kidney of *H. signifer* coded for a protein of 440 amino acids, and the molecular mass of Gulo (50.8 kDa) from *H. signifer* was comparable to that (50.96 kDa) from *Scyliorhinus torazame* [38]. In general, Gulo/GULO amino acid sequences contain several strongly hydrophobic regions which may be associated with the endoplasmic reticulum membrane [39]. Indeed, Gulo/GULO are concentrated in the microsomal fraction during cellular fractionation, indicating that they are membrane-bound [40], [41]. Similarly, the Gulo from *H. signifer* comprised two transmembrane regions, indicating that it is an integral membrane protein.

The deduced amino acid sequence of Gulo from *H. signifer* contained two conserved domains, the FAD-binding domain at positions 21–156 and the ALO domain at positions 180–428. Since Gulo/GULO is an oxygen-dependent oxidoreductase using FAD as a co-factor, the FAD binding domain includes a covalent FAD-binding site, with a conserved His54 residue to which FAD binds via an 8-alpha-(N3-histidyl)-riboflavin linkage [41]. During the catalytic reaction, flavin first undergoes reduction by accepting two electrons from the substrate L-gulono-γ-lactone, followed with oxidation by O_2_ which presumably involves the ALO domain. The process produces H_2_O_2_ and 2-oxo-L-gulono-γ-lactone, which forms L-ascorbic acid after non-enzymatic isomerisation [42].

Cho et al. [15] analysed Gulo/GULO from fish and mammals and reported that a significant number of amino acid residues were exclusive to either mammals or fishes. An example would be the Pro98 residue in mammalian GULO versus Glu98 in fish Gulo. However, the Gulo from *H. signifer* did not conform to amino acid residues exclusive to fish, and it contained several amino acid residues in the conserved regions not found in Gulo/GULO of other animals. These amino acids included Ser28, Lys42, Gln76 and Ile103 in the FAD-binding domain, and Ala185, Leu319, Glu325 and Lys382 in the ALO domain. In spite of these replacements, the Gulo of *H. signifer* is phylogenetically closer to elasmobranchs than to bony fishes and amphibians. It shares the highest percentage similarity, in a descending order, with those of *T. scyllium*, *S. torazame* and *M. manazo*. Among the residues found only in *H. signifer*, Ser28 could be related to the regulation of Gulo in this stingray because it is a potential phosphorylation site located in the FAD-binding domain. Furthermore, analysis of the phosphorylation and glycosylation sites across the Gulo/GULO of different species (Table 2) revealed that those from the euryhaline freshwater *H. signifer* and the stenohaline freshwater *Potamotrygon motoro* possess the least number of phosphorylation sites. These findings indicate that successful invasion of the freshwater environment could have induced changes in the post-translational regulation of Gulo activity in the kidney of elasmobranchs.

### Salinity changes and oxidative stress

In aquatic organisms, salinity change causes a variety of physiological responses such as increases in plasma concentrations of stress-related hormones, stimulation of energy metabolism, and perturbation of steady state concentrations of electrolytes. It has been established that salinity-induced stress is associated with increased production of reactive oxygen species, causing oxidative damage. Liu et al. [43] reported that acute salinity stress caused changes in activities of superoxide dismutase, catalase, glutathione peroxidase in shrimp (*Litopenaeus vannamei*). In addition, they [43] reported that the resistance of *L. vannamei* to acute salinity changes could be enhanced by moderate doses of dietary supplement of vitamin E. For fish, Roche and Boge [20] reported changes in antioxidant enzymes in the red blood cells of sea bass (*Dicentrarchus labrax*) subjected to hypoosmotic shock. Choi et al. [44] demonstrated that salinity changes could lead to changes in the mRNA expression of glutathione peroxidase and glutathione-S-transferase in the olive flounder (*Paralichthys olivaceus*). They [44] concluded that these two enzymes played an important role in the detoxification of reactive oxygen species and might serve as indicators of salinity-induced oxidative stress in *P. olivaceus*. Furthermore, Martínez-Alvarez et al. [21] reported that acclimation from freshwater to seawater resulted in salinity-induced oxidative stress in the sturgeon (*Acipenser naccarii*) as reflected by increases in activities of catalase, glutathione peroxidase and superoxide dismutase, and in lipid peroxide concentrations, in blood, liver and heart.

In comparison, the concentrations of thiobarbituric acid reactive substances remained statistically unchanged in the gills and liver of *H. signifer* after 1 day or 6 days of transfer form freshwater to brackish water. Hence it can be concluded that acclimation from freshwater to brackish water did not lead to increased oxidative stress in *H. signifer*. However based on thiobarbituric acid reactive substances alone, it would be difficult, if not impossible, to judge whether *H. signifer* would experience less salinity-induced oxidative stress in brackish water. Since thiobarbituric acid reactive substances represent terminal products of lipid peroxidation [45], an increase in oxidative stress would lead to their accumulation, but a reduction in oxidative stress does not necessarily result in an increase in their removal. Indeed, after 20 days of exposure to seawater, plasma osmolality, erythrocyte constants and muscle water content in *A. naccarii* return to levels comparable to those of the freshwater control [21]. This would mean that successful acclimation from freshwater to seawater has been achieved, leading to a reduction in salinity stress caused by seawater exposure. However, the concentration lipid peroxidation products did not return to control level [21].

### Why would acclimation from freshwater to brackish water lead to decreases in renal *gulo* mRNA expression and Gulo activity in *H. signifer*?

Molecular phylogeny suggests that freshwater stingrays from Southeast Asia, including *H. signifer*, have only marine species as their nearest relative, and they underwent multiple colonizations rather than speciations within freshwater [46]. Since the invasion of the *H. signifer* lineage into freshwater habitats appears to be relatively recent [47], it is not unexpected that *H. signifer* would experience higher salinity stress in freshwater than in brackish water. Indeed, freshwater *H. signifer* has to enter a brackish or marine environment at some point in its life history, possibly for reproduction [23]. Unlike the stenohaline freshwater *P. motoro, H. signifer* is ureogenic and ureotelic in freshwater [24]. Although *H. signifer* down-regulates the ornithine-urea cycle capacity and reduces the retention of urea in freshwater, its plasma urea concentration (43.8 mmol l^−1^) is much higher than that of *P. motoro* (0.65 mmol l^−1^). Consequently, in freshwater, the blood plasma osmolality of *H. signifer* (416 mosmol kg^−1^) is considerably higher than that of *P. motoro* (349 mosmol kg^−1^) [24]. In addition, urea concentrations in muscle, liver and brain of freshwater *H. signifer* (70.9, 49.4 and 59.3 µmol g^−1^ wet mass, respectively) are much higher than those of *P. motoro* (0.38, 0.04 and 0.38 µmol g^−1^ wet mass, respectively) [24]. Consequently, *H. signifer* would experience greater osmotic stress in freshwater than in brackish water due to the steep ionic and urea gradients between the blood and the environment. After transfer from freshwater to brackish water, *H. signifer* would logically experience a decrease in osmotic stress and hence a decrease in salinity-induced oxidative stress, leading to a significant decrease in renal *gulo* mRNA expression and Gulo activity.

In rodents, exposure to xenobiotics leads to increased oxidative stress, and there are increases in ascorbate concentrations in tissues and urine due to up-regulation of ascorbic acid biosynthesis [48], [49]. In mouse [50], but not rat [48], exposure to xenobiotics also leads to increases in *GULO* expression and GULO activity in the liver. In addition, a negative feedback of ascorbic acid intake on the synthesis of GULO has been reported in mouse [51]. Although Moreau et al. [52] reported that Chondrostei fish showed unchanged Gulo activity with increasing dietary ascorbic acid, the slow rate of ascorbic acid synthesis in poikilotherms may account for this observation. Subsequently, Moreau and Dabrowski [53] reported that an increase in dietary levels of α-tocopherol and/or ascorbic acid significantly raised the concentrations of these two antioxidants in the liver, and concomitantly lowered the Gulo activity in the kidney, of the white sturgeon *Acipenser transmontanus*. Being potent free radical scavengers, α-tocopherol and ascorbic acid could affect the overall redox state of the cell, which in turn modulated the level of Gulo expression and activity in the white sturgeon [53]. Therefore, like mammals, changes in redox status can lead to changes in *gulo*/Gulo expression in fish. Since decreases in renal *gulo* mRNA expression and Gulo activity in *H. signifer* exposed to brackish water indicate a decrease in the production of ascorbic acid, and since salinity-induced oxidative stress has been demonstrated in fish (sea bass, [20]; sturgeon, [21]; olive flounder [44]), it can be deduced that *H. signifer* was in an improved redox status, probably due to a reduction in salinity-induced oxidative stress after transfer from freshwater to brackish water.

### Changes in tissue ascorbate and/or dehydroascorbate concentrations indicate that *H. signifer* encounters less salinity-induced oxidative stress in brackish water

The concentration of ascorbate + dehydroascorbate in the kidney of *H. signifer* (∼43 µg g^−1^ wet mass) falls in the range of 37–123 µg g^−1^ wet mass as reported for a variety of fishes [54], [55]. The steady state concentration of ascorbate in the kidney is maintained by a balance between its synthesis through Gulo and its oxidation to dehydroascorbate plus its transport out to the blood. Results on *gulo* mRNA expression and Gulo activity indicate a decrease in ascorbate production in the kidney of *H. signifer* exposed to brackish water, but yet the concentrations of total ascorbate + dehydroascorbate, ascorbate and dehydroascorbate in the kidney remained unchanged. Hence, it can be deduced that there was a decrease in the overall demand of ascorbate as an antioxidant in non-renal organs/tissues of *H. signifer* during brackish water acclimation, corroborating the proposition that *H. signifer* experienced less salinity stress in brackish water than in freshwater.

Ascorbate is a vital antioxidant in the mammalian brain [4]. It is transported into the brain and neurons via sodium-ascorbate co-transporter 2, and accumulated within these cells against a concentration gradient [56]. The highest concentration of ascorbate in the human body is found in the brain (∼2–10 mmol l^−1^) and in neuroendocrine tissues such as adrenal, and it is difficult to deplete ascorbate from the brain [57]. Since human cannot synthesize ascorbic acid, a high brain ascorbate concentration is attributed to (a) an adequate dietary supplementation of vitamin C, (b) effective ascorbate/dehydroascorbate transport mechanisms [58], and (c) efficient reducing systems to maintain the vitamin in the form of ascorbate [59], [60], [61], [62]. Ascorbate probably also serves as an important antioxidant in fish brains because high brain ascorbate concentrations have been reported for *Oplegnathus fasciatus* (254 µg g^−1^) [63] and *Anguilla japonica* (251 µg g^−1^) [64]. In addition, the brain shows the slowest rate of decline in ascorbate concentration when teleost fishes, which cannot synthesize ascorbic acid, were subjected to dietary vitamin C deficiency [65], [66]. Similarly, a high concentration of ascorbate + dehydroascorbate was detected in the brain of *H. signifer* (approximately 222 µg g^−1^ wet mass), with much higher concentration of ascorbate than dehydroascorbate. Therefore, it is logical to deduce that the brain of *H. signifer* possesses transport mechanisms for ascorbate and/or dehydroascorbate, and biochemical mechanisms/systems to maintain ascorbate in the reduced state. Since there were a significant increase and a significant decrease in the concentrations of ascorbate and dehydroascorbate, respectively, with the total ascorbate + dehydroascorbate concentration remained unchanged in the brain of *H. signifer* after 1 day of exposure to brackish water, it can be concluded that brackish water acclimation indeed led to a decrease in oxidative stress in the brain.

The gills of freshwater elasmobranchs play an important role in osmoregulation [22], and are presumably one of the major epithelia that are directly exposed to oxidative stress due to environmental disturbances. In comparison, the concentration (15 µg g^−1^ wet mass) of ascorbate + dehydroascorbate in the gills of *H. signifer* was much lower than those in the brain and kidney. It is possible that besides ascorbate, other antioxidant mechanisms such as superoxide dismutase, catalase and glutathione could also play an important role in branchial oxidative defence [67]. There were significant increases in concentrations of ascorbate and total ascorbate + dehydroascorbate, with the dehydroascorbate concentration remained unchanged, in the gills of *H. signifer* after exposure to brackish water for 1 or 6 days. Since *gulo* was not expressed in the gills, it would imply that there was an increase in the uptake of ascorbate from the blood, and that the excess ascorbate was not converted to dehydroascorbate due to a decrease in salinity-induced oxidative stress when *H. signifer* was acclimated from freshwater to brackish water. If indeed *H. signifer* would experience increased oxidative stress in freshwater, the accumulation of ascorbate in the gills during brackish water acclimation can be viewed as a possible preparation for it to readjust back to freshwater when opportunity arises.

### Conclusions

*H. signifer* has a short history of freshwater invasion [47] and is apparently confronted with greater salinity stress in freshwater than in brackish water [24]. Our results demonstrate for the first time that brackish water acclimation could lead to a down-regulation of renal *gulo* expression and a decrease in renal Gulo activity, indicating a decrease in the capacity of ascorbic acid synthesis, in *H. signifer*. Yet, concentrations of ascorbate increased in some organs, which in some cases were accompanied with a decrease in dehydroascorbate concentration, indicating that *H. signifer* indeed experienced lower salinity-induced oxidative stress in brackish water. Taken altogether, these results suggest that the ability to upregulate ascorbic acid production to combat hyposalinity-induced oxidative stress could have contributed in part to the successful invasion of the freshwater habitat by some euryhaline marine elasmobranchs.
